# Continuous glucose monitor accuracy during extracorporeal membrane oxygenation

**DOI:** 10.1016/j.ccrj.2023.11.003

**Published:** 2023-12-13

**Authors:** Tipwarin Phongmekhin, Ray Wang

**Affiliations:** Department of Medicine, University of Melbourne, Grattan Street, Parkville, Victoria, 3010, Melbourne, Australia; Department of Medicine, University of Melbourne, Grattan Street, Parkville, Victoria, 3010, Melbourne, Australia; Department of Diabetes and Endocrinology, Royal Melbourne Hospital, 300 Grattan Street, Parkville, Victoria, 3052, Melbourne, Australia

**Keywords:** Continuous glucose monitor, Critical illness, ECMO, ICU, Type 1 diabetes

The use of continuous glucose monitors (CGMs) in critically ill patients is an evolving area of research. Limited information is known about the accuracy of CGM in critically ill patients requiring extracorporeal membrane oxygenation (ECMO).

We present the case of a man aged in his mid-50s who required ECMO whilst using a Dexcom G6 CGM device. His medical history included type 1 diabetes mellitus with most recent HbA1c of 7.0% (53 mmol/mol), prior coronary artery bypass graft, hypertension, and dyslipidaemia.

He presented to hospital with central chest pain, bradycardia, and hypotension. After commencing an epinephrine infusion, his electrocardiogram demonstrated a wide complex tachycardia with left bundle branch block pattern (not meeting Sgarbossa's criteria), and chest X-ray revealed acute pulmonary oedema. The patient subsequently experienced a pulseless electrical activity cardiac arrest. After 47 min of cardiopulmonary resuscitation failed to restore spontaneous circulation, venoarterial ECMO was instituted. Balloon angioplasty was performed on an occluded native left anterior descending the artery after stent insertion attempts via percutaneous coronary intervention were unsuccessful. Additional percutaneous coronary intervention to an 80–90% stenosed mid-distal right coronary artery was successful.

Post angiography, he was admitted to the intensive care unit (ICU) on a norepinephrine infusion (4–20mcg/min) for blood pressure control. His diabetes was managed with an intravenous insulin infusion (IVII). Table 1 shows CGM glucose paired with corresponding arterial blood gas glucose results whilst receiving ECMO. The patient was successfully decannulated after 3 days of ECMO and was eventually discharged home after a prolonged admission for heart failure management.

**Table 1:** Paired continuous glucose monitor and arterial blood gas glucose results.

Day of admission	Time (24 h format)	SBP/DBP (MAP) (mmHg)	ABG glucose (mmol/L)	CGM glucose (mmol/L)	% absolute relative difference
1	21:25	101/68 (78)	17.0	15.4	9.4
1	22:26	89/66 (74)	15.7	15.1	3.8
3	12:54	100/60 (76)	14.0	15.5	10.7
3	14:59	94/61 (71)	13.5	15.3	13.3
				MARD	9.3 %

ABG, arterial blood gas; CGM, continuous glucose monitor; DBP, diastolic blood pressure; MAP, mean arterial pressure; MARD, mean absolute relative difference; SBP, systolic blood pressure.

CGM can be a reliable tool for in-hospital glucose measurement with comparable accuracy to traditional glucose-testing methods.^1^, ^2^, ^3^ A study using a Dexcom G6 CGM system demonstrated a mean absolute relative difference value of 12.1% when comparing CGM to laboratory values in 153 critically ill patients, one of whom was on ECMO (though details regarding that patient are not reported).^3^ We found a mean absolute relative difference value of 9.3% recorded by the Dexcom G6 CGM whilst our patient received ECMO (Table 1), though few paired values were obtained limiting the generalisability of these results. This was likely a result of transmitter–reader Bluetooth connectivity issues whilst the patient was in the ICU with personal belongings secured away. Low tissue perfusion may have also contributed to intermittent transmission of CGM data, whether due to hypotension or mechanical compression of the CGM device (positioned on his posterior upper arm) whilst supine in the ICU.^4^ The diabetes team was unaware of the patient during his ICU admission, when interventions to ensure connectivity and data transmission could have provided greater CGM data whilst receiving IVII in the ICU. Studies have shown that CGM use may allow ICU nurses to easily monitor glucose levels of patients receiving IVII, reducing time and equipment needed for blood glucose monitoring.^5^ We report the first detailed description of a case of CGM use on ECMO, suggesting CGM may be a reliable tool in monitoring glucose levels even in critically ill patients such as those on ECMO, though studies with more data points are needed.

## Informed consent statement

Not applicable as no patient-identifying details were included.

## CRediT authorship contribution

TP wrote the initial manuscript draft. RW revised the manuscript and provided supervision.

## Funding

This research did not receive any specific grant from funding agencies in the public, commercial, or not-for-profit sectors.

## Conflict of interest

The authors declare that they have no known competing financial interests or personal relationships that could have appeared to influence the work reported in this paper.
